# Improving medication adherence in stroke patients through Short Text Messages (SMS4Stroke)-study protocol for a randomized, controlled trial

**DOI:** 10.1186/s12883-015-0413-2

**Published:** 2015-08-28

**Authors:** Ayeesha Kamran Kamal, Quratulain Nauman Shaikh, Omrana Pasha, Iqbal Azam, Muhammad Islam, Adeel Ali Memon, Hasan Rehman, Muhammad Affan, Sumaira Nazir, Salman Aziz, Muhammad Jan, Anita Andani, Abdul Muqeet, Bilal Ahmed, Shariq Khoja

**Affiliations:** Neurology, Stroke Service, The International Cerebrovascular Translational Clinical Research Training Program (Fogarty International Center, National Institutes of Health) and Department of Medicine, Aga Khan University, Karachi, Pakistan; The International Cerebrovascular Translational Clinical Research Training Program (Fogarty International Center, National Institutes of Health) and Aga Khan University, Karachi, Pakistan; Director Masters in Epidemiology and Biostatistics Program, Department of Community Health Sciences, Aga Khan University, Karachi, Pakistan; Department of Community Health Sciences, Aga Khan University, Karachi, Pakistan; SMS4Stroke Study, The International Cerebrovascular Translational Clinical Research Training Program (Fogarty International Center, National Institutes of Health) and Aga Khan University, Karachi, Pakistan; Department of Medicine, Aga Khan University, Karachi, Pakistan; Stroke Service, Section of Neurology, Department of Medicine The International Cerebrovascular Translational Clinical Research Training Program (Fogarty International Center, National Institutes of Health) and Aga Khan University, Karachi, Pakistan; eHealth Innovation, Global, Aga Khan Development Network, Karachi, Pakistan; Epidemiology and Biostatistics, Department of Medicine, Aga Khan University, Karachi, Pakistan; Evidence, Capacity & Policy mHealth Alliance, United Nations Foundation Geneva Associate Professor COMSATS Institute of Information Technology, Islamabad Visiting faculty-University of Calgary, Calgary, AB Canada

**Keywords:** Stroke, Medication adherence, SMS, Prevention

## Abstract

**Background:**

Stroke is a major cause of morbidity and mortality, especially in low and middle income countries. Medical management is the mainstay of therapy to prevent recurrence of stroke. Current estimates are that only 1 in 6 patients have perfect adherence to medication schedules. Using SMS (Short Messaging Service) as reminders to take medicines have been used previously for diseases such as diabetes and HIV with moderate success. We aim to explore the effectiveness and acceptability of SMS in increasing adherence to medications in patients with stroke.

**Methods:**

This will be a randomized, controlled, assessor blinded single center superiority trial. Adult participants with access to a cell phone and a history of stroke longer than 1 month on multiple risk modifying medications will be selected from Neurology and Stroke Clinic. They will be randomized into two parallel groups in a 1:1 ratio via block technique with one group receiving the standard of care as per institutional guidelines while the parallel group receiving SMS reminders for each dose of medicine in addition to the standard of care. In addition intervention group will receive messages for lifestyle changes, medication information, risk factors and motivation for medication adherence. These will bemodeled on Social Cognitive Theory and Health Belief Model and will be categorized by Michies Taxonomy of Behavioral Change Communication. Patient compliance to medicines will be measured at baseline and then after 2 months in each group by using the Morisky Medication Adherence Scale. The change in compliance to medication regimen after the intervention and the difference between the two groups will be used to determine the effectiveness of SMS reminders as a tool to increase medication compliance. The acceptability of the SMS will be determined by a tool designed for this study whose attributes are based Rogers Diffusion of innovation theory.

A sample size of 86 participants in each arm will be sufficient to detect a difference of 1 point on the MMAS with a power of 90 % and significance level of 5 % between the two groups; using an attrition rate of 15 %, 200 participants in all will be randomized.

**Discussion:**

The SMS for Stroke Study will provide evidence for feasibility and effectiveness of SMS in improving post stroke medication adherence in an LMIC setting.

**Trial registration:**

https://clinicaltrials.gov/ct2/show/NCT01986023 11 /11/2013

**Electronic supplementary material:**

The online version of this article (doi:10.1186/s12883-015-0413-2) contains supplementary material, which is available to authorized users.

## Background

Stroke is a major cause of death and disability worldwide that affects 16 million persons annually [1]. The burden is especially high in low and middle income countries which account for more than 85 % of all stroke related mortality [2, 3]. Pakistan is no different, with a lifetime stroke prevalence of 19 % and a recurrence risk of 53 % in the first year alone [4–7].

Long term mortality due to stroke is most frequently related to cardiovascular disease (cerebrovascular or heart disease) [8, 9]. Predictors include diabetes mellitus, atrial fibrillation, large artery atherosclerosis and ischemic heart disease [10–14], all of which are modifiable through medical management. Effective therapy for stroke hence comprises of a combination of antiplatelet, anti-lipid and antihypertensive therapy [15]. For medical management to be effective however, compliance to medication schedule is the single most important factor.

Current estimates are that only one sixth of all patients come close to perfect adherence to their medication regimen [16]. The issue is gaining importance due to an increase in the prevalence of chronic diseases and an aging population [17]. Common causes of medication non adherence include complex treatment regimens [18] and patient's lack of belief in the treatment [19]. Practical ways to improve medication adherence are still not well established.

Strategies that have previously shown success include telephone calls, behavior therapy and dissemination of information materials relating to adherence [20–22]. These interventions however are often complex and require abundant resources which make them impractical in resource strapped regions [20]. Using innovation and technology offers alternative solutions that may also be able to address the issue of cost and resources. One such solution is the use of the Short Text Message system (SMS). In the last 10 years, there has been an exponential increase in the use of mobile phones in Pakistan. There are currently more than 132 million active mobile phone connections in the country as compared with a little over 5 million in 2004 [23]. This translates to around 77 % of all Pakistanis [23] having access to a cell phone today and with it, SMS. The rise has been due to extremely affordable costs. An SMS in Pakistan costs around 0.27 cents [24]. Use of SMS is thus a potential tool in aiding patients adhere to medication schedules.

SMS has been used previously in diseases such as asthma, HIV and diabetes with variable success [25–36]. However, to the best of our knowledge, tailored SMS for each patient based on their dosing frequency, have not been studied before. Moreover SMS to promote health, that are informed by Health Theory and coded by Taxonomy for replicability, are new and in line with developing the science of informed replicable mHealth interventions [37].

We hypothesize that sending SMS as medication reminders for each dose and promoting healthy behavior will improve medication adherence among stroke patients This trial therefore aims to assess this effect of SMS on patient compliance to medication, by customizing the SMS frequency for each individual according to his/her dosing frequency. The secondary objective is to assess the acceptability and patient satisfaction associated with this intervention. The trial will also attempt establish if an intervention like this is cost effective in a low and middle income setting.

## Methods

### Design overview (Fig. 1 (Study flow chart))

The SMS4stroke trial will be a randomized, controlled, assessor blinded single center superiority trial. The participants will be randomized into two parallel groups in a 1:1 ratio via block technique with one group receiving the standard of care as per institutional guidelines while the parallel group receiving SMS reminders for each dose of medicine in addition to the standard of care. The intervention group will also receive twice weekly SMS that reinforce healthy behaviors and the importance of taking medications.

**Fig. 1:**
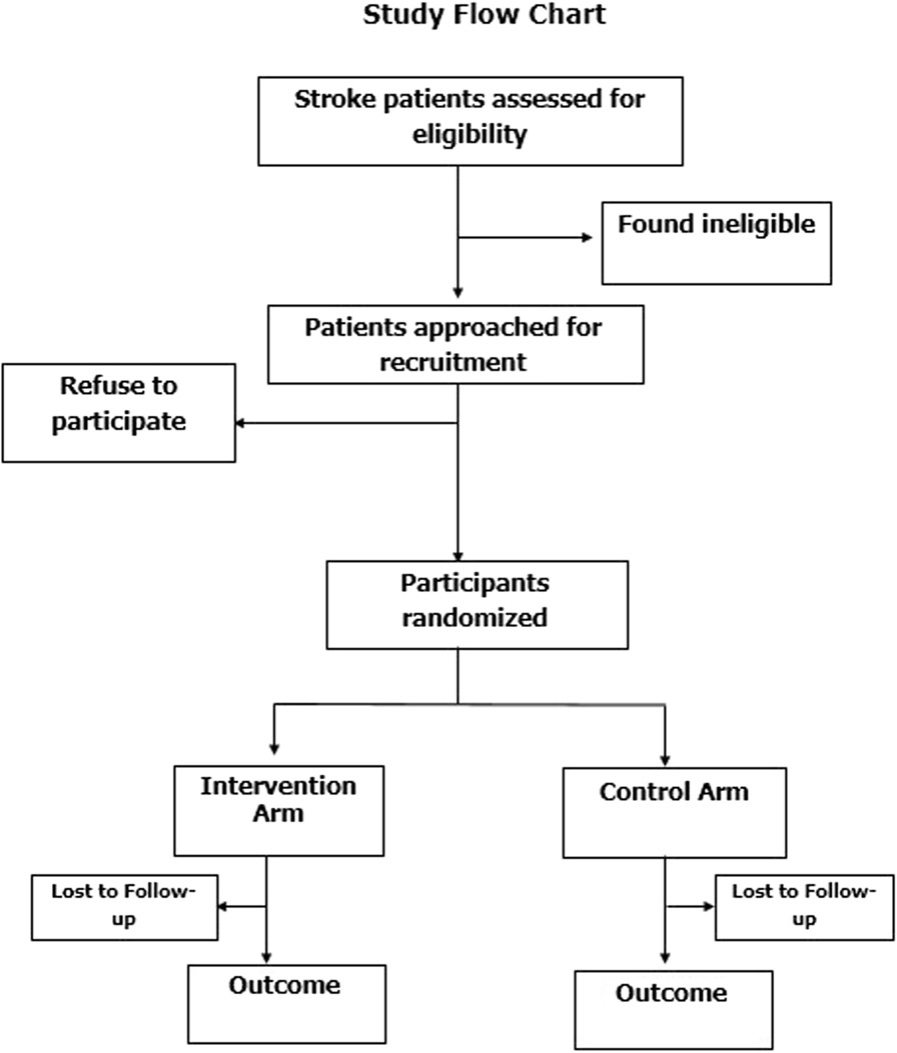
Study Flow Chart

**Fig. 2:**
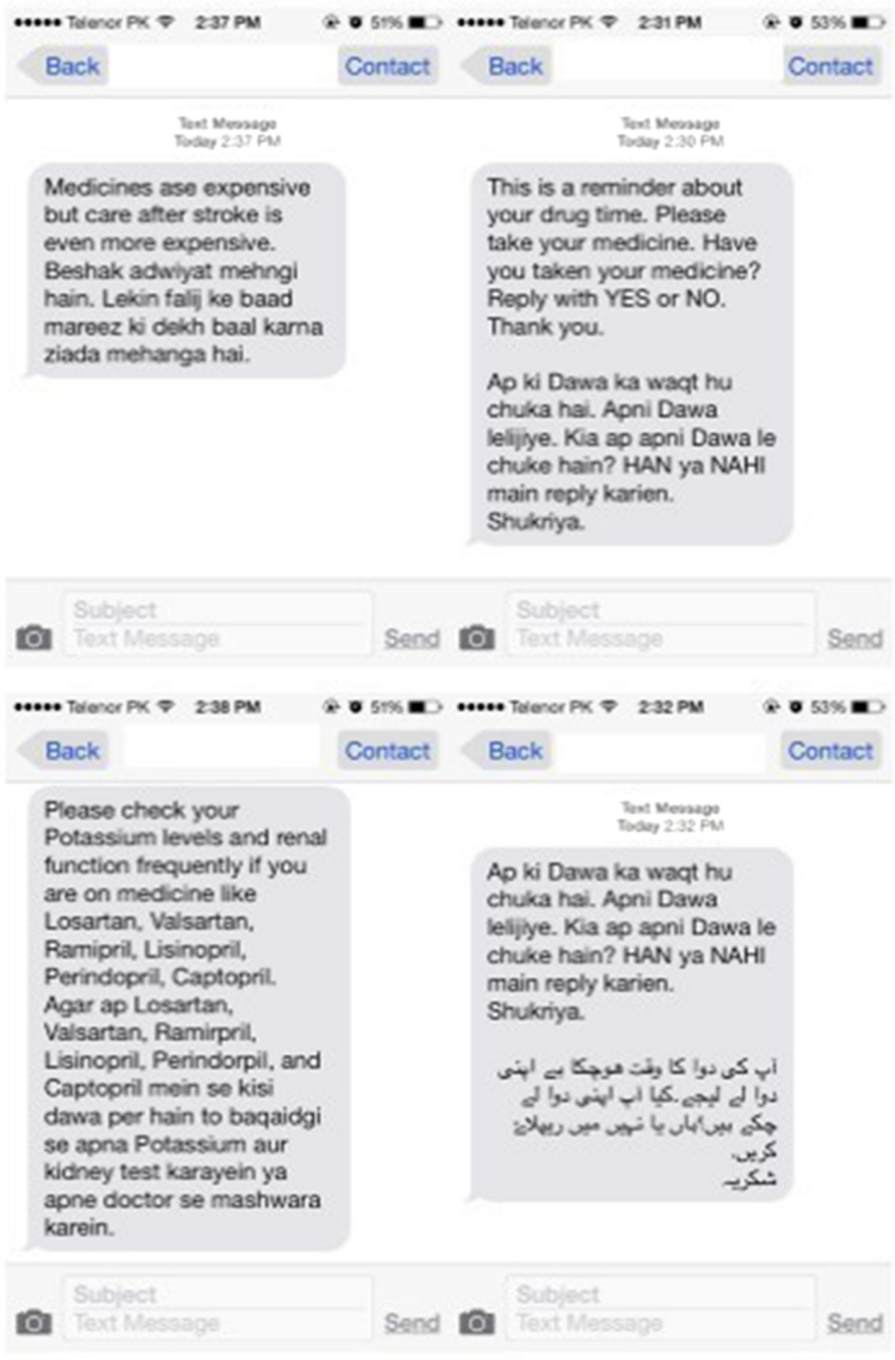
Screenshots of SMS (Templates)

### Study setting

The trial will be conducted in the Neurology & Stroke Clinic at the Aga Khan University Hospital (AKUH), Karachi, Pakistan. The center is a Joint Commission International Accreditation (JCIA) accredited tertiary care hospital located in Karachi, a large metropolitan city located in the south of Pakistan. Stroke service is delivered through a 24 h emergency neurovascular team and ambulatory care clinics. The hospital caters to a large and diverse population with an estimated 100 patients attending Neurology & Stroke Clinic each day. The average annual stroke clinic volume is 1500 patient visits. The center also has logistic and technical expertise available from dedicated research support staff and an independent Clinical Trials Unit (CTU) and a dedicated Aga Khan Development Network e-Health Resource Center.

### Participants

Participants will be selected from patients who present to the Neurology and Stroke Clinic at the center.

#### Inclusion criteria

- Age greater than 18 years old
- History of stroke(s) confirmed by neuroimaging at the time of the episode
- >1 month since last episode of stroke
- Use of at least two drugs such as (but not limited to) anti platelets, statins, anti-hypertensives to control risk factors of stroke.
- Modified Rankin Score of 3 or less.
- Possession of a personal cell phone that the patient has access to at all times. In the case of patients who do not own or are unable to use mobile phones, they must have a caregiver available at all times who possesses a cell phone.
- Ability to receive, comprehend and reply to an SMS in English, Urdu or Roman Urdu. In the case of patients who themselves are unable to receive, comprehend or reply to an SMS, they must have caregivers available at all times who could perform the above mentioned tasks.

In addition all participants will be required to provide a written, informed consent prior to enrollment Additional file 1.

#### Exclusion criteria

- Biological impairment in reading or responding to SMS such as (but not limited to) loss of vision, visual field cuts, aphasia.
- Diagnosed organ dysfunction or malignancy such as liver failure, renal dysfunction etc. that require frequent medication changes.
- Plans to travel outside the country inside the two months following enrollment and during SMS intervention with a SIM unable to receive text messages.

### Interventions

#### Control group

In the control group, patients will receive the usual standard of care provided at the center for stroke patients. This primarily consists of regular follow up visits (frequency as per patient needs) with their stroke neurologist. The standard schedule for clinic visits after an uncomplicated recovery is 2 weeks, 1 month, 3 months and 6 months after index stroke. On each clinic visit, a detailed neurological examination is performed, stroke risk factors are assessed and concerns and queries are addressed. In addition, the patient is counseled regarding beneficial life style modifications and the significance of compliance with medication regimens. Reading material about their disease in English or Urdu is offered to each patient. None of these activities however are extended beyond their clinic visit. Each patient is, however, provided with a telephone number that can be used to reach the stroke team in case of an emergency and each patient is also reminded of their clinic appointments 1–2 days prior via SMS and/or phone as per center policy.

#### Intervention group

In addition to the standard of care as provided to the control group, automated SMS serving as reminders for patients to take their medicines will be sent to each patient in the intervention group. These SMS will be sent at the time patients are scheduled to take their medications, i.e. once, twice or thrice daily as advised by their physician. The text will be sent for each dose once, collectively for all drug groups; hence some patients might receive multiple SMS in a day. The participants will be required to respond to the SMS stating whether or not they have taken their medicines for that time. The patient response will be recorded. No action will be taken if the patient does not reply to an SMS or if the patient responds saying that they have not taken the medicine. The patient will be reached via phone call if he/she fails to respond for at least 5 consecutive days to identify the reason.

Furthermore, SMS will be sent twice weekly to each patient in the intervention group that reinforces the importance of taking medications and other healthy behaviors. These SMS will be customized to each patient’s risk profile and medications by the research team. For example, some of these SMS will be drug specific i.e. they explain the benefit of a particular drug and so they will only be sent to those taking that drug. The SMS will be phrased using Michie et al’s taxonomy for behavior change interventions [38]. The health information messages will cover information about disease, modifiable risk factors, drug information and importance of optimal drug adherence. These are 16 different messages, tailored to individual patients and will be sent over an 8 week period twice weekly. The set of messages for each patient will be selected at the time of recruitment and then sent at the scheduled timings by our IT staff. These messages will change during the duration of the study for the participant based on their profile for e.g. a non-smoking participant is not to get a message about quitting smoking. However for the overall study these are standardized messages that have been worded and coded carefully.

Using the standardized definitions as presented in the taxonomy allows for easy reproducibility and generalized applicability for future work related to this intervention. The specific content of the SMS is derived based on the Social Cognitive theory [39] and Health Belief model [40] of behavior change in health care. Templates used include “Stroke can happen again. Don’t forget to take your medicine” and “High blood pressure can cause stroke. Please take your blood pressure medicine.” The purpose of adapting this model for designing SMS is to make them effective in communicating to the participants the consequences of their behavior on health (Fig. 2).

Additional file 2 provides details of these SMS along with the taxonomy number for each SMS design. The definitions of each taxonomy are also provided in the appendix.

The Frontline SMS software will be used to send the SMS. The software has open access and is free to use. Additional file 3 describes the software usage for this specific study.

### Outcomes

The primary outcome of interest will be a change in medication adherence after 2 months of receiving the SMS. These findings will be compared to its corresponding findings from the control group to account for the Hawthorne effect.

Medication adherence will be measured at recruitment and after 2 months in both groups on the Morisky Medication Adherence Scale [18]. The scale has been used in a similar setting previously and it has been translated and validated in Urdu [41]. The change will be determined by comparing the score on the MMAS before and after the intervention.

The instrument consists of 8 questions and the response to each question is scored as either 0 or 1. The maximum score, hence, through this questionnaire is 8. A higher score indicates better adherence. A score of < 2 indicates low adherence.

Secondary outcome measures include patient satisfaction and acceptability of using an e-health based innovation such as the SMS to improve clinical outcome. This will be done through a tool that has been designed for IT based interventions and adapted for this study. This tool identifies beneficial and untoward attributes of the SMS for stroke intervention by users. The tool design for measuring acceptability of innovation is based on Roger's Diffusion of Innovations theory. The tool hence has questions that specifically assess the relative advantage of SMS, its compatibility with the study population, its complexity, its trialability and observability [42].

### Participant timeline

Recruitment will begin with signing of an informed consent and assigning the patient an ID number. Data collection will begin with a detailed interview regarding the demographic and clinical details of the patient including his/her prescription details. The baseline MMAS score for each patient will also be calculated at this time. This will be followed by the research staff leaving the room and a senior investigator (QS) taking over at which point the patient will be randomized to either the intervention or the control group by the independent CTU staff.

If the patient is allocated to the intervention arm, the investigator (unblended) will explain the details of the intervention and will demonstrate by sending one test SMS on his/her cell phone (in English and Urdu both).

If there is a change in patient prescription with respect to any drug or dose by any physician, the patient will be requested to notify at a 24 hours helpline number answered by trained staff. In addition, since participants are tagged in the hospital system as trial participants all visits are logged and there will be tracking to ascertain any changes in prescription which will be medically confirmed.

Following the recruitment visit, the patients will not be required to come back to clinic for any additional visits for the purposes of this study for 2 months at which time there will be a follow up interview. At this time, patient adherence will be assessed by the research officer again who will be blinded.

### Duration

The trial will continue till achievement of sample size and 2 months follow up for outcome ascertainment thereafter.

### Sample size

Based on literature, we estimated the mean MMAS score to be 6 [43] in the control arm and 7 in the intervention group, giving a mean difference of 1. A standard deviation of 2 is assumed for this score. Using these values, a sample of at least 172 subjects will be required to achieve a power of 90 % and significance level of 5 % when testing a two tailed hypothesis. This translates into 16 % effect size as hypothesized. Keeping a 15 % attrition rate the sample size was inflated so that at least 100 subjects will be targeted for each arm in the sample.

### Assignment of interventions

Cards with patient assignments to either the intervention or the control group will be prepared by the CTU through computerized block randomization technique in block sizes of 10. This is to ensure similarity between the two groups at all times permitting interim analysis during the study. Additionally block size was not discussed with the research team interacting with participant recruitment to limit predictability of assignment and researcher bias.

The randomization cards will be sealed in opaque envelopes, marked by ID and kept with the CTU. Even if the envelope is held against bright sunlight one will not be able to ascertain the allocation. After enrollment, obtaining informed consent and completing the baseline measurements, the CTU will be informed and randomization of the participant will be done.

### Data collection methods

The patients will be asked to come in 2 months later for a follow up interview to assess the outcome of the trial. Data will be collected on a Data collection form (DCF) in this interview by a research officer who will be blinded to the allocation of the participants. This primarily consists of the MMAS and a tool that assesses patient satisfaction regarding the SMS innovation.

Participants in both arms will be contacted 2 days prior to interview as reminders for their appointment. They will be compensated for their fare for reaching the center. In case, the participants do not report for their follow up appointment, they will be contacted and their appointments will be rescheduled.

### Pilot testing

Pilot Testing will be performed on 10 % of the sample size i.e. 20 patients before commencement of the study. As per the actual study, participants will be recruited from Neurology and Stroke Clinics and standard procedures will be followed. This will allow testing the intervention for smooth application and any systematic errors. Problems (if any) with eligibility criteria will be identified and the feasibility of achieving sample size will be assessed. The data collection tool will be assessed for flow of questions and comprehensiveness. The intervention will be assessed for practicality relating to delivery, accuracy, timeliness, comprehension and satisfaction. These participants will be excluded from the final study sample.

### Data management

Research officers will be specifically trained to collect all data during patient interview leaving no data collection forms incomplete. All data collected will be sent to the research office in sealed envelopes. At the end of each day, any missing data on forms will be noted. In case, of missing data, the participant will be contacted to fill the missing information. The data will be entered by a separate team that is formed for data entry on Microsoft Access. This team does not include any members involved with research. As per institutional policy, double entry is mandatory for all data. The data entry operators will be blinded to the allocation of each patient. All data entered will be backed up centrally.

### Statistical methods

Analysis will be performed using the intention to treat principle at two stages: interim analysis after 25 % of the sample has been reached and final analysis after data has been collected from all study participants. The analysis will be reported to the project team who will publish the results in a timely manner. Descriptive statistics will be reported as mean (with standard deviation) for continuous variables like age, years of schooling, years since diagnosis, MMAS score etc. Proportions will be reported for categorical variables like gender, marital status, area of residence, employment status, proportion of patients with depression etc.

The primary endpoint will be the difference in medication adherence level between the two groups as detected by the MMAS after 2 months of the intervention.

The acceptability and patient satisfaction of the intervention will be reported through proportions with a break up for Roger’s factors. In addition, we will be monitoring the study for participant ‘alert fatigue’ and withdrawals from the intervention.

All data analysis will be performed on STATA version 12. We plan to apply regression modeling techniques to control for confounders or other covariates such as baseline clinical status as calculated by the Modified Rankin Scale, patient age, the severity of their stroke, number of medications, caregiver or stroke survivor cell phone user are all important variables that will be included in the data analysis as to how they affect adherence. Sensitivity analyses are planned for caregiver SMS recipient vs. direct users for response rates to the intervention.

### Ethics and human subjects protection

All patients taking part in the trial will be required to provide written informed consent at the time of recruitment. Consent forms will be approved by the ERC and they will be available in English and Urdu. The participants will have the right to withdraw at any moment during the study; their inclusion or exclusion from the study will not affect the usual standard care provided to them. In addition, all participants will be provided financial reimbursement for all costs pertaining to follow up visits and replying to reminder SMS. Participants identified with depression and dementia will be referred to appropriate physicians for further management.

In addition, special care will be taken to maintain the confidentiality of the patient's disease. The name of the institution sending the SMS will not be mentioned in the SMS, nor will be the patient’s name. In addition, special care will be taken to send the health promotional texts twice weekly at times that do not cause discomfort to the patient such as late at night.

In days of high security alerts in the city and announced SMS blackouts, it is planned to send advance SMS the night before to the participant that there would not be any SMS the next day to avoid participant anxiety or uncertainty about continuity of care or reception of services.

All staff has received Good Clinical Practice (GCP) training and a face to face training in a 2 day Neuroethics course regarding research in the developing world where all design aspects for this trial were discussed.

The study is approved by the Ethical Review Committee of the Aga Khan University, Karachi, Pakistan. The study Ethical Review Number is 2763-Med-ERC-13. Any changes to the protocol need to be approved by the ERC before implementation. Monthly reviews on the progress of the trials will be conducted by the ERC including inspection of signed informed consent forms. Although the trial is intended to last around 3 months, in case trial duration exceeds 6 months, the ERC will require the protocol to be resubmitted for review.

### Access and dissemination policy

Only the PI and research team will have access to the study results who will be responsible for publishing in a timely manner. The funding source will not have any special access or privileges regarding results before publication. However since this study is funded by NIH , all publications will be open access in PubMed Central as per NIH policy [44].

The ERC and the CTU will be provided access if required. Safety issues and adverse events will be reported to the ERC of the Aga Khan University.

Results will be disseminated through scientific journals, conferences and meetings. They will be available at https://clinicaltrials.gov as soon as they are finalized. They will also be shared with concerned departments and patients.

## Discussion

This study protocol presents the design of a randomized controlled trial using SMS for improving medication adherence in stroke patients.

SMS has been used to increase medication adherence to HIV medications, polio vaccination and maternal and child care populations with most of the interventions in first world populations.

There are several unique aspects to this study. Firstly, SMS has never been used in a unique high risk stroke population living in resource strapped settings with marginal support and poor health literacy. Secondly, the messages themselves are not just simple knowledge transfer texts, the wording and the content has been designed by the Health Belief Theory and Social Cognitive Model. Thirdly, each communication message is informed by taxonomy and coded for easy replicability of intervention. Fourthly, we use interoperable replicable open access software. Fifth, each message is highly personalized, tailored to the risk profile, habits and medication prescription of each participant. Sixth, our study protocol, design and methods are robust by study design standards [45, 46] of centralized randomization, allocation concealment, blinded assessment, intention to treat analysis, design to ensure maximum follow up and minimize attrition, staff training and endpoint assessment.

The major limitation of this study is the choice of measure of primary outcome i.e. self-report of medication adherence on Morisky Medication Adherence Questionnaire. Although this tool has been validated in Urdu in the country, using assessment tools such as biochemical markers or electronic pill boxes may potentially provide more accurate results. This however isn’t actually possible because of the complexity of the prescription of a stroke patient; it includes drugs for multiple risk factors and multiple drugs at a time. Simply opening an electronic pill box registers a dose as administered even if the patient takes only two medicines out of the four he was prescribed for that time thus an electronic pill box couldn’t capture the data of a complicated regimen. Running biochemical tests for the each of these medications would be costly and impractical and require repeat hospital visits and participant fatigue. Also, the rationale of IT based intervention in SMS is to cue behavior at a distance and ensure adherence, getting patients to make repeat visits during intervention and interact with the caregiver may then be more responsible for adherence than the intervention itself. In addition to this rationale, we had logistic and resource limitations to consider when we designed this study and repeated pill count visits by disabled stroke survivors and caregivers was not feasible in the realm of this current protocol. Considering all these pros and cons, we opted for a reply SMS when the medication was taken by the participant and the self-reported MMAS. Another limitation is that this study is designed to study the effect of SMS on adherence, a longitudinal study to report the change in risk factors via SMS like decrease in blood pressure, cholesterol and control of glucose, or even a change in endpoint like prevention of stroke or myocardial infarction would further the science of Information Technology based intervention. Furthermore, we acknowledge that we do not know mechanistically which component of the SMS intervention will have the effect of improving adherence. However, we targeted the SMS intervention to cover both intentional non adherence (due to lack of knowledge, or myths, or attitude, which is the educational SMS) and unintentional non adherence (due to cognitive issues, forgetfulness etc., which is the reminder SMS). These issues are discussed in the clinic but there is usually not enough time and not enough reminders and presence to reinforce these messages, thus we have not aimed to target one kind of SMS over the other, but to try to increase adherence via SMS by targeting both components of non-adherence through SMS.

This study will be regionally important. Pakistan, like other emerging low and middle income countries, faces non communicable diseases like stroke as its major health challenge, so a cheap intervention would have high impact. The population is uniquely high risk for stroke given its profile, one in three is hypertensive, one in four diabetic, with rising rates of depression and suicidality. Currently the country has an electronic database with a unique identifier number, thus the potential of reaching mass population for NCD primary prevention as an extension is possible. Since most out of pocket catastrophic expenditure on stroke pushes most Pakistanis below poverty line, when simple prevention would have helped, even if the effect size is modest, in a population dense area, SMS will still save countless lives. Future studies should focus on using SMS to improve clinically meaningful outcomes like reduction of blood pressure, control of dyslipidemia and reduction of recurrent stroke through effective secondary prevention mediated by improved adherence to medications.

### Trial status

Ongoing

## Additional files

Additional file 1:
**Data Collection Form.** (DOCX 44 kb) Additional file 2:
**SMS Text and Coding.** (DOCX 30 kb) Additional file 3:
**Frontline SMS software.** (DOCX 1021 kb)

## Abbreviations

SMS: Short text message
AKUH: Aga Khan University Hospital
JCIA: Joint Commission International Accreditation
CTU: Clinical trials unit
MMAS: Morisky medication adherence scale
